# Understanding COVID-19 Vaccine Acceptance among Latin American Health Workers: Implications for Designing Interventions

**DOI:** 10.3390/vaccines11091471

**Published:** 2023-09-10

**Authors:** Tamara Rivera, Jennifer Brustrom, Maite Vera Antelo, E. Benjamin Puertas, Dale A. Rhoda, Martha Velandia-Gonzalez

**Affiliations:** 1 Comprehensive Immunization Program, Pan American Health Organization, 525 23rd Street, NW, Washington, DC 20037, USA; 2 Biostat Global Consulting, 330 Blandford Drive, Worthington, OH 43085, USAdale.rhoda@biostatglobal.com (D.A.R.); 3 Human Resources for Health Unit, Pan American Health Organization, 525 23rd Street, NW, Washington, DC 20037, USA

**Keywords:** COVID-19, vaccination, vaccines, immunization, health personnel, Americas

## Abstract

Health workers (HWs) have a key role in promoting vaccine acceptance. This study draws on the Behavioral and Social Drivers of Vaccination (BeSD) model and our team’s investigation of vaccine hesitancy in a sample of 1197 HWs across 14 Caribbean countries in 2021. We conducted a cross-sectional Internet survey of 6718 HWs across 16 countries in Latin America in spring 2022, after the COVID-19 vaccine had recently become widely available in the region. The survey assessed HWs’ attitudes regarding COVID-19 vaccines and vaccines in general. As a proxy measure of COVID-19 vaccine acceptance, we used the willingness to recommend the COVID-19 vaccine to eligible people. Ninety-seven percent of respondents were COVID-19 vaccine acceptant. Although nearly all respondents felt that the COVID-19 vaccine was safe and effective, 59% expressed concerns about potential adverse effects. Despite uniformly high acceptance of the COVID-19 vaccine overall and across Latin American subregions, acceptance differed by sex, HW profession, and COVID-19 history. Social processes, including actions and opinions of friends, family, and colleagues; actions and opinions of religious leaders; and information seen on social networks shaped many respondents’ opinions of vaccines, and the magnitude of these effects differed across both demographic and geographic subgroups. Information campaigns designed for HWs should underscore the importance of vaccine safety. Messages should be tailored to specific audiences according to the information source each is most likely to consult and trust.

## 1. Introduction

Although COVID-19 vaccination has saved tens of millions of lives worldwide [1], vaccination coverage varies tremendously across regions, with many countries falling far short of the World Health Organization’s (WHO) vaccination coverage target of 70% [2]. Even among health workers (HWs), vaccination coverage is not universal. Studies across the globe have shown COVID-19 vaccine acceptance rates among HWs ranging from 21% (Egypt) to 95% (Asia-Pacific) [3,4], and averaging only 63% [5] and 69% [6] in surveys of HWs from multiple countries. 

Demographic characteristics consistently associated with COVID-19 vaccine acceptance among HWs worldwide include sex, age, race/ethnicity, and profession, with many studies indicating that males [3,5,6,7], older HWs [3,5,6], those who identify as white, and physicians are more likely to accept the COVID-19 vaccine relative to their counterparts [3,5,7,8]. History of receiving the seasonal flu vaccine is also highly correlated with COVID-19 vaccine acceptance among HWs [3,4,5,8]. Globally, the three most consistently cited COVID-19 vaccine-related concerns among HWs are vaccine safety, effectiveness, and potential side effects [3,9]. 

### Context and Study Overview

**Figure 1:**
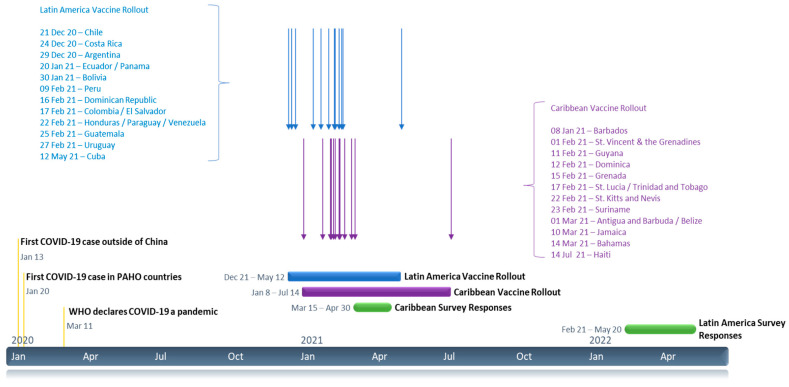
Timeline of COVID-19, vaccine rollout, and survey data collection in Latin America and the Caribbean [16,17,18].

The toll of the COVID-19 pandemic on HWs in the Americas has been particularly high [10]. Vaccines, which first began to be rolled out in Latin America in December 2021 (Figure 1), played a critical role in controlling the spread of the disease. As of 31 December 2022, 28 million HWs in Latin America and the Caribbean had had at least one dose of COVID-19 vaccine and 24 million had completed the vaccine series [11]. 

This paper reports on a cross-sectional Internet survey of 6718 health care workers from 16 countries in Latin America conducted after the COVID-19 vaccine had recently become widely available in the region (Figure 1). 

The purposes of this project were the following:

- To estimate the percentage of Latin American HWs who were accepting of the COVID-19 vaccine at the time of the survey.
- To explore whether there were differences in the magnitude of COVID-19 vaccine acceptance among demographic and geographic subgroups of the study population.
- To explore demographic predictors of COVID-19 vaccine acceptance in Latin American HWs.
- To relate the findings of the project survey to those of our earlier survey of HWs in the Caribbean [12,13] in order to inform vaccination-related information dissemination and education campaigns for HWs.

The survey instrument and methodology for this study were based on the Behavioral and Social Drivers of Vaccination (BeSD) model, which posits that vaccine uptake is influenced by four domains: what people think and feel about vaccines (thinking and feeling), social processes that favor or inhibit vaccination (social processes), individual motivations (or reluctance) to seek vaccination (motivation), and practical issues that affect seeking and accepting vaccination (practical issues) [14]. The study also draws on lessons learned from our team’s investigation of vaccine hesitancy in a sample of 1197 HWs across 14 Caribbean countries in the spring of 2021 [12,13] (Figure 1). To our knowledge, our studies are the largest theoretically based studies of HW vaccine acceptance during the COVID-19 era in Latin America and the Caribbean conducted to date. Vaccine acceptance among HWs is critical because HWs’ attitudes to vaccines influence their own vaccine uptake and the probability that they will recommend the vaccine to their own patients [15].

## 2. Materials and Methods

### 2.1. Survey Instrument 

Items for the survey instrument were drawn from the BeSD of COVID-19 vaccination tool [19] and a questionnaire developed by researchers at the University of California, Los Angeles (UCLA) [20], and were employed in the earlier Caribbean survey [12,13]. The survey was subsequently adapted for use in Latin America in collaboration with experts from the Pan American Health Organization’s (PAHO’s) Department of Health Systems and Services (Human Resources for Health Unit) and Comprehensive Immunization Program. This included its augmentation with several questions regarding respondent background and opinions regarding vaccines. The final survey instrument contained three multiple choice and four yes/no questions assessing respondent demographics and background, including COVID-19 history, availability of COVID-19 vaccines, and COVID-19 vaccination history. General attitudes towards vaccines, willingness to be vaccinated, reasons for delaying or refusing COVID-19 vaccines, factors contributing to opinions of COVID-19 vaccines, and attitudes towards flu/influenza and hepatitis B vaccines were measured with 34 Likert items assessing agreement with each item (four-point scale ranging from strongly agree to strongly disagree). Respondents were invited to share additional information about their vaccine-related opinions and experience through six optional open-ended items. The survey was pilot-tested with 13 participants from eight different Latin American countries prior to being fielded. The survey was conducted in Spanish. The Spanish survey instrument and an English translation are provided in Supplementary Materials, Files S1 and S2, respectively. 

### 2.2. Sample Size

The minimum target sample size for each country was set at 200 to estimate the percentage of HWs who accept the COVID-19 vaccine (two-sided Wald-type 95% confidence level not wider than +/− 7% in each country even if acceptance were as low as 50%). 

The sample size calculation took into account the total number of HWs in the categories reported to the World Health Organization (WHO) portal of National Health Workforce Accounts (NHWA; e.g., nurses, midwives, doctors, pharmacists, and dentists). Sixteen Latin American countries which provide data on human resources for health to NHWA, including seven from the Central America subregion (Costa Rica, Cuba, Dominican Republic, El Salvador, Guatemala, Honduras, and Panama) [12,13], five from the Andean Community subregion (Bolivia, Colombia, Ecuador, Peru, and Venezuela), and four from the Southern Cone (Argentina, Chile, Paraguay, and Uruguay), reported a total of 1,928,776 HWs (405,520 from Central America; 680,695 from the Andean Community; and 842,561 from the Southern Cone).

### 2.3. Survey Implementation

Representatives from PAHO and national ministries of health promoted the survey through announcements distributed by private health and academic institutions, scientific and professional associations, private practices, and independent professionals. Each respondent accessed the Internet survey using a quick response (QR) code. Individuals who met the inclusion criteria (age 18 or older and identified as a HW) and consented were enrolled in the survey. Data were collected anonymously using the Qualtrics^®^ survey platform.

Survey data in Cuba were collected primarily with paper questionnaires because of barriers inhibiting prospective respondents’ access to the Qualtrics^®^ platform. The questionnaire was distributed in approximately 50 health institutions in 15 municipalities of Havana. Ministry of Health personnel distributed and collected the paper questionnaires. 

No payments or incentives were given for completing the survey. Data were collected between 21 February and 20 May 2022.

### 2.4. Analysis

Data were analyzed using Stata 17 (StataCorp. 2021. Stata Statistical Software: Release 17, StataCorp LLC., College Station, TX, USA). In the aggregate analysis, countries served as strata. 

Responses to the demographic and opinion questions were summarized using proportions. Data were reported as if they were from a simple random sample of HWs in each country (within-country design effect = 1). When combining results across countries, each country’s response was weighted by the number of doctors and nurses there, according to the WHO NHWA portal. 

Responses to each of the 34 Likert-style opinion questions were summarized using four categories—strongly agree, agree, disagree, strongly disagree—and two combined categories: strongly agree and agree (agree); disagree and strongly disagree (disagree). The main analysis approach was to look for patterns in responses between respondent categories across groups of questions. Rao–Scott survey-adjusted chi-square tests were conducted to identify statistically significant associations.

We used responses to the item *I would recommend the COVID-19 vaccine to eligible people* (Question #14d) as a proxy measure of COVID-19 vaccine acceptance. Those who agreed or strongly agreed with this statement were classified as COVID-19 vaccine acceptant; those who disagreed or strongly disagreed with this statement were regarded as not acceptant. Because most HWs in Latin America were required to receive a COVID-19 vaccine as a condition of employment, we felt that willingness to recommend the vaccine better captured vaccine acceptance than vaccine uptake [21]. In the context of the survey, “eligible people” were those eligible to receive the COVID-19 vaccine per the criteria established by each country’s ministry of health, including front-line HWs, older adults, people with comorbidities that increased their risk of severe illness, and pregnant people. 

Each chi-square analysis considered the association between an opinion question and a single respondent characteristic. Multivariable logistic regression was employed to assess associations while controlling for six respondent characteristics. Responses of strongly agree or agree were coded 1 and those of disagree or strongly disagree were coded 0. Independent variables for the regression included sex (two levels; females are the reference group), age quartile (reference = youngest quartile), HW category (five levels; reference = physicians), ethnicity (three levels; reference = white), workplace (four levels; reference = public), and history of previous COVID-19 diagnosis (two levels; reference = yes).

To identify patterns in regression results, figures were used to summarize the percentage of respondents who selected agree or strongly agree across six sets of demographic subgroups, and figure cells were highlighted (a) if covariate levels had a statistically significant logistic regression coefficient and (b) if the percentage of respondents in that category who agreed with the statement was at least 10 percentage points higher or lower than the percentage in the reference category. 

The data and chi-square and logistic regression results for all 34 questions and all respondent categories are provided in the final project report [22]. 

### 2.5. Summary of Free-Response Questions 

The survey instrument contained a total of nine free-response questions. The study team classified responses to these questions by thematic category and, later, by BeSD category. Details of the qualitative coding are provided in the final report [22].

## 3. Results

In this section, we describe respondent characteristics and summarize measures of vaccine acceptance and attitudes and opinions towards vaccines. A report containing the comprehensive survey results is available online [22].

### 3.1. Respondent Characteristics

A total of 6718 HWs from across 16 countries responded to the survey (2855 from Central America; 2390 from the Andean Community; and 1473 from the Southern Cone). Every country reached the target of at least 200 respondents, with a range of sample sizes from 207 to 1056 (Supplementary Materials File S3). 

**Figure 2:**
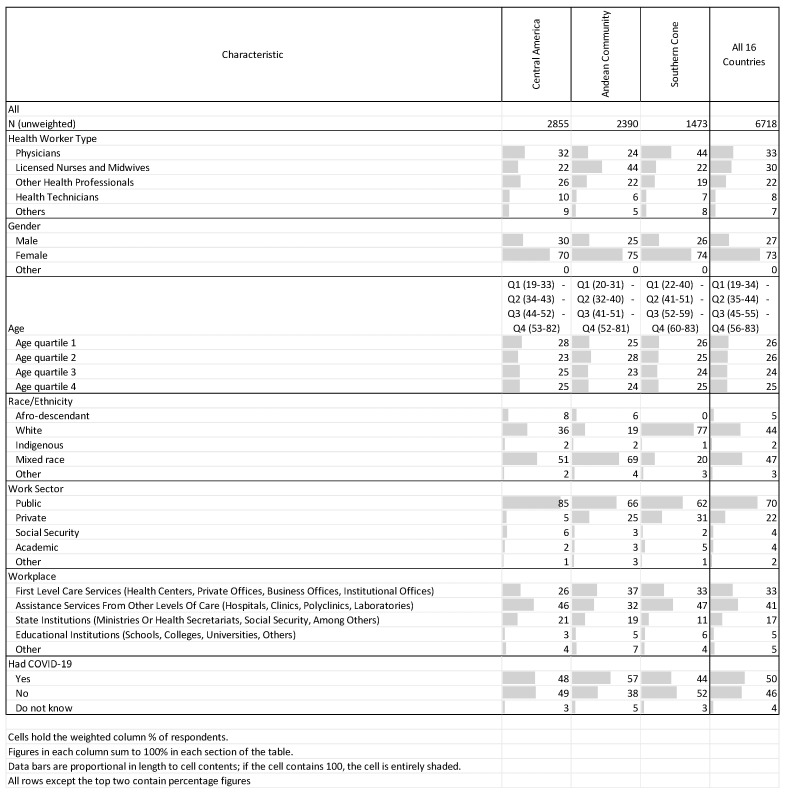
Respondent demographic characteristics and COVID-19 history, by region and across all countries combined.

In Figure 2, respondent demographic characteristics and COVID-19 history are presented by region and across all countries combined. Most respondents were either physicians (33%) or nurses (30%), and the sample was predominantly female (73%). The youngest quarter of respondents were aged between 19 and 34 years and the oldest quarter were aged between 56 and 83 years. Most respondents self-identified as either mixed race (47%) or white (44%), and the majority worked in the public sector (70%). Nearly three-quarters of respondents worked in either assistance services (41%) or first-level-care services (33%), and exactly half reported that they had had COVID-19 sometime before participating in the survey.

Ninety-nine percent of respondents reported ever having been vaccinated for COVID-19, and 98% reported that they had the complete vaccine series (i.e., single dose and booster dose/additional dose in any schedule; first dose, second dose, and booster dose/additional dose in any schedule; or two booster/additional doses in any schedule (single dose or two doses)). Access to COVID-19 vaccination services was also almost universal. Among the 318 individuals who provided a reason for not having had the complete COVID-19 vaccination schedule by the time they completed the survey, the most common reasons for refusing or delaying the vaccine were concerns about side effects/adverse reactions (22%), illness- or health-related reason other than COVID-19 (14%), and a perception of unreliable scientific evidence (14%). 

### 3.2. Vaccine Acceptance

**Figure 3:**
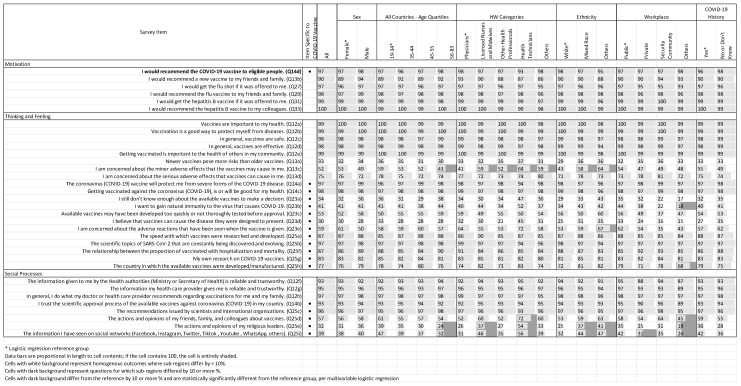
COVID-19 vaccine acceptance and vaccination attitudes and opinions, by respondent demographic characteristics and COVID-19 history. Source: [22], data reprinted with permission.

**Figure 4:**
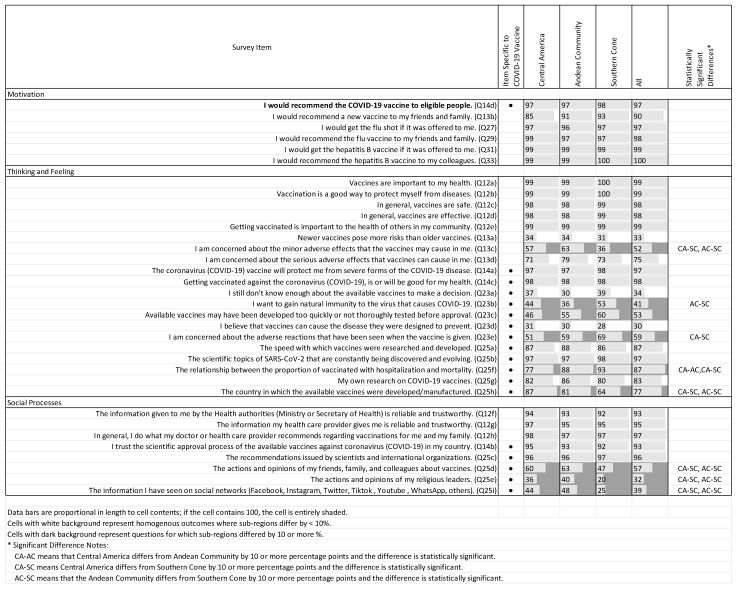
COVID-19 vaccine acceptance and vaccination attitudes and opinions, by Latin American subregion.

Figure 3 summarizes responses relating to attitudes and opinions regarding vaccines across all countries, stratified by respondent demographic characteristics, including sex, age quartile, HCW category, ethnicity, and workplace, as well as COVID-19 history. Figure 4 summarizes responses stratified by Latin American subregion (Central America, Andean Community, and Southern Cone). In both figures, items are organized by BeSD category, and cells are shaded when two conditions are met: (a) the difference between the category and the reference category is statistically significant according to multivariable logistic regression and (b) that difference is more than 10 percentage points. 

COVID-19 vaccine acceptance was almost universal among the respondents in our sample, with 97% agreeing or strongly agreeing with the statement “I would recommend the COVID-19 vaccine to eligible people” (Figure 3 and Figure 4).

Nearly all respondents also indicated that they would recommend the flu/influenza vaccine to their friends and family (98%), and all stated that they would recommend the hepatitis B vaccine to their colleagues. Ninety percent of respondents reported that they would recommend a new vaccine to their friends and family (Figure 3 and Figure 4).

There were no statistically significant differences among any of the demographic or geographic subgroups for any of the motivation survey items.

### 3.3. Attitudes and Opinions Regarding Vaccines

#### 3.3.1. Thinking and Feeling

Overwhelmingly, respondents felt that the COVID-19 vaccine was safe and effective: 97% agreed or strongly agreed with the statement “The COVID-19 vaccine will protect me from severe forms of the COVID-19 disease”, and 98% agreed or strongly agreed that “getting vaccinated against COVID-19 is or will be good for my health”.

Fifty-nine percent of our sample agreed or strongly agreed that they were concerned about potential adverse effects associated with receiving the COVID-19 vaccine, with respondents in the other ethnicity category being more likely to express this opinion than individuals who self-reported as white (67% vs. 53% of respondents, respectively) (*p* = 0.044). Concern about possible adverse effects of the COVID-19 vaccine were more common among respondents from the Southern Cone relative to those in Central America (69% vs. 51%, *p* < 0.001).

#### 3.3.2. Social Processes

Many respondents reported that their attitudes and opinions regarding vaccines were influenced by the actions and opinions of friends, family, and colleagues (57% of respondents); actions and opinions of religious leaders (32%); and information seen on social networks (39%). The magnitude of these effects differed across both demographic and geographic subgroups. For example, health technicians reported being influenced by the actions and opinions of friends, family, and colleagues to a greater extent than physicians (72% vs. 52%, *p* < 0.001), as did nurses (60%, *p* < 0.001). Individuals from “other” workplaces were much less likely to be influenced by these factors relative to those who work within a public workplace (45% vs. 58%, *p* = 0.016). Respondents in the Southern Cone reported being less influenced by friends, family, and colleagues (47%) than those in Central America (60%, *p* < 0.001) or the Andean Community (63%, *p* < 0.001).

Religious leaders’ actions and opinions had a greater influence on the youngest respondents compared to the oldest respondents (39% vs. 24%, *p* < 0.001), and on licensed nurses and midwives (37%) and health technicians (54%) relative to physicians (26%, both *p* < 0.001). Similarly, these leaders had a greater reported effect on individuals of self-reported mixed race (37%) and others (41%) relative to white respondents (25%, both *p* < 0.001), and on respondents working in the public sector compared to those in other work sectors (35% vs. 19%, *p* = 0.001). Respondents in the Southern Cone reported being less influenced by religious leaders (20%) than those in either Central America (36%, *p* < 0.001) or the Andean Community (40%, *p* < 0.001).

Self-reported influence of social media also differed across respondent groups. The youngest respondents were much more likely to agree or strongly agree that the information they saw on social networks influenced their opinion of COVID-19 vaccines compared to the oldest respondents (47% vs. 32%, *p* = 0.006). Nurses (46%) and health technicians (56%) were more likely to report being influenced by social media compared to physicians (31%, both *p* < 0.001). Similarly, respondents who self-identified as mixed race (44%) and belonging to other ethnicities (47%) mentioned being more influenced by social networks compared to those who self-identified as white (32%, both *p* < 0.001). Finally, health personnel working in the public sector were more influenced by social media compared to those working in the private sector and in other sectors (42%, 31%, and 26%, respectively, both *p* < 0.001). Individuals in the Southern Cone (25%) were less influenced by social media than their counterparts in either Central America (44%, *p* < 0.001) or the Andean Community (48%, *p* < 0.001).

#### 3.3.3. General Opinions of Vaccines

Respondents were nearly unanimous in their agreement that vaccines are generally safe and effective. However, three-quarters of respondents expressed concern about potential serious adverse effects associated with vaccines in general, and this pattern did not differ statistically across demographic or geographic categories. Further, slightly more than half of respondents (52%) were concerned about mild adverse effects related to vaccines in general, and the level of concern differed across several respondent categories. Relative to respondents in the youngest age quartile (ages 19–34), the oldest respondents (ages 56–83) were less likely to be concerned about minor side effects (43% vs. 59%, respectively; *p* = 0.016). Compared to physicians (41%), 59% of nurses, 52% of other health professionals, 68% of health technicians, and 59% of others reported agreeing or strongly agreeing that they were concerned about minor side effects associated with vaccines in general (all *p* < 0.001). Compared to those who self-reported their ethnicity as white (43%), those of mixed race (58%) and others (64%) were more likely to be concerned about minor adverse effects (all *p* < 0.001). Concerns about minor adverse effects also differed by subregion, with those in the Southern Cone (36%) less likely to be concerned relative to those in Central America (57%, *p* < 0.001) or the Andean Community (63%, *p* < 0.001).

#### 3.3.4. Vaccine Acceptance: Bivariate and Multivariate Analyses

**Figure 5:**
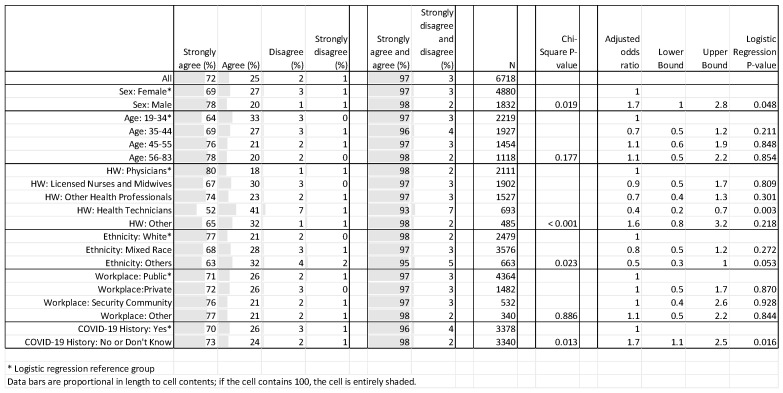
COVID-19 vaccine acceptance, as assessed by agreement with the statement “I would recommend the COVID-19 vaccine to eligible people” (Q14d)—all countries: associations with respondent demographic characteristics.

Figure 5 shows acceptance of the COVID-19 vaccine, both overall and stratified by sex, age, HW job-type, ethnicity, workplace, and COVID-19 history. From left to right, results are categorized across four agreement levels (i.e., whether respondents strongly agreed, agreed, disagreed, or strongly disagreed with the statement “I would recommend the COVID-19 vaccine to eligible people”), and the next two columns show results categorized across two agreement categories (strongly agree and agree vs. strongly disagree and disagree) and the results of chi-square analyses. The right-most section of the figure shows the results of the multivariable logistic regression.

Logistic regression indicated that, holding the other demographic variables constant, males were more likely than females to be vaccine acceptant (OR = 1.7, 95% CI [1, 2.8], *p* = 0.048); health technicians were less likely than physicians to be vaccine acceptant (OR = 0.4, 95% CI [0.2, 0.7], *p* = 0.03), and those who had not had COVID-19 (or who did not know whether they had had it) were more vaccine acceptant than respondents who had had COVID-19 (OR = 1.7, 95% CI [1.1–2.5], *p* = 0.016).

## 4. Discussion

This study explored COVID-19 vaccine acceptance among Latin American HWs who responded to an internet survey between February and May 2022. At the time the survey was conducted, the COVID-19 vaccine had recently become widely available in the region (Figure 1). Findings from this study, coupled with results from our earlier survey of HWs in the Caribbean [12], have important implications for informing and designing communication campaigns related to vaccines and vaccination and for implementing capacity-building activities targeted towards HWs.

### 4.1. Key Findings

#### 4.1.1. Vaccine Acceptance

In this study of Latin American HWs, 97% of respondents reported being acceptant of the COVID-19 vaccine (as assessed by their response to whether they would recommend the COVID-19 vaccine to those eligible to receive it), and 98% reported that they had had the complete COVID-19 vaccination schedule. This acceptance rate is higher than the acceptance rate observed in an earlier survey of HWs in Latin America and the Caribbean [23] and also than that observed in our 2021 survey of Caribbean HWs [12,13] (acceptance rates of 83% and 77%, respectively).

In our study, physicians were relatively more acceptant of the COVID-19 vaccine than health technicians, which is consistent with what we observed among Caribbean HWs [12,13]. In contrast to the Caribbean study, in which nurses were less acceptant than physicians, there was very little difference in the COVID-19 vaccine acceptance levels of nurses and physicians in the Latin American sample. Although the youngest respondents in the Caribbean study were significantly less acceptant of the COVID-19 vaccine relative to older respondents [12,13], there were no significant differences in acceptance levels among Latin American respondents.

Interestingly, in both of our studies, the patterns of COVID-19 vaccine acceptance were identical to patterns of flu/influenza vaccine acceptance (97% acceptance of both vaccines in the Latin American sample; 77% acceptance of both vaccines in the Caribbean sample). This finding suggests that COVID-19 vaccine acceptance may be reflective of sentiments toward other vaccines recommended for HWs, and it is consistent with other research [3,4,5].

#### 4.1.2. Vaccine Safety and Effectiveness, and Concerns about Side Effects

Despite strong agreement that vaccines are safe and effective, approximately three-quarters of respondents in the Latin American and the Caribbean studies [12,13] expressed concern about the potential serious adverse effects caused by vaccines. Further, more than half of Latin American respondents reported being concerned about minor side effects that vaccines may cause, and more than half were specifically concerned about the adverse reactions observed in individuals who had taken the COVID-19 vaccine. Our findings are consistent with other studies demonstrating that concern about potential adverse reactions to a COVID-19 vaccine is one of the main reasons for vaccine hesitancy among HWs [3,9].

#### 4.1.3. Influence of Social Processes

Findings from our studies of Latin American and Caribbean HWs [12] underscore the substantial role that social influences can play in shaping individuals’ opinions of vaccines. Both studies also provide evidence that some groups are more susceptible to social influence than others. In both studies, for example, the youngest respondents were most likely to report being influenced by social media [12]. Further, among Latin American HWs, health care technicians, non-white respondents, and those who work in the “other” sector were more likely to report being influenced by the actions and opinions of religious leaders and by the information they have seen on social networks. Respondents in both Central America and the Andean Community reported being more influenced by friends, family members, and colleagues; religious leaders; and social media, relative to their peers in the Southern Cone. Our results are consistent with other studies showing the importance of social media [24] and religious leaders [25] in influencing attitudes towards vaccination.

### 4.2. Recommendations

The findings of this study and our past work in the Caribbean [12,13] have several notable implications for designing information dissemination and education campaigns for HWs, namely the following:

#### 4.2.1. Integration with Essential Public Health Functions

It is important to consider the essential public health functions framework to provide a comprehensive approach to vaccine acceptance among HWs in the region, which should include policy and legislation development, health promotion, access to services (including vaccination), development of human resources for health, social participation, and health financing.

#### 4.2.2. Emphasize Safety in Information Campaigns

Given that concerns about vaccination-related adverse effects was a significant source of concern among the respondents in this study and others [3,8,11,12], it is important that information campaigns designed for HWs continue to underscore the importance of vaccine safety. According to health communication experts, one of the most effective ways to relay this kind of information to all audiences is to use principles of risk communications and community engagement (RCCE) to provide accurate, transparent, and timely information regarding the risks and benefits of vaccine-preventable diseases and vaccines themselves [26]. While it is important for new HWs to receive proper education regarding vaccines, vaccination, and immunization, it is also most important for countries to invest in the continuous education of HWs to ensure that information regarding the development of new vaccines is imparted from legitimate channels of information. Finally, continuing to monitor the sentiments of HWs regarding vaccines and immunizations—old and new, routine and pandemic—is a key activity needed to continue to understand HWs’ perceptions and sentiments and adapt interventions and messaging campaigns accordingly.

#### 4.2.3. Ensure Messages Are Delivered through Trusted and Preferred Information Channels

The subgroup differences we observed highlight the need to deliver messages to specific audiences according to the information source they are most likely to consult and trust. For example, young individuals, nurses and midwives, non-white respondents, and those who work in the public sector might be more inclined to respond to vaccination promotion messages that come from faith leaders. These same groups of individuals might also be more likely to be persuaded by vaccination-related messages on social media platforms.

#### 4.2.4. Educate HWs to Be Critical Consumers of News and Other Information

Although HWs may be less likely than the general public to be persuaded by vaccine-related misinformation [27], it is crucial to educate HWs to consume news and other information critically so that they know what information to believe, share, and utilize. Individuals should be taught to identify the source of the information they encounter and the motive of the messenger relaying the information, and to then discern whether the information is verifiable [28,29]. On social media platforms, knowledgeable HWs may have the opportunity to help combat misinformation and address vaccine hesitancy [30] and should be encouraged to do so.

### 4.3. Strengths and Limitations

The strengths of this study are as follows:

- The study was publicized widely in medical networks.
- The survey was available online for three months.
- Workers at 50 health facilities in Cuba were afforded the opportunity to complete a paper version of the questionnaire.
- Pre-testing helped clarify questions for respondents.
- The survey and protocol were fine-tuned in accordance with our experience studying COVID-19 vaccine hesitancy among HWs in the Caribbean [12,13].
- Seventy-three percent of respondents were female, which is reflective of the composition of the health workforce in Latin America [31].

The study also has several limitations:

- The survey findings correspond to a single point in time, and the results cannot be projected into the future.
- Participants were a convenience sample of Latin American HWs. The respondents were limited to those who heard of the study, had the capability to participate online, and decided to do so. The sample is not likely to be representative of all HWs in any Latin American country or subregion, nor does it have respondents from Mexico or Brazil. For this reason, the results should not be generalized to all HWs in Latin America.
- The open invitation to participate was circulated through numerous professional networks, but it is not possible to know what portion of HWs heard about the survey in time to participate, nor whether those who learned about the survey are similar in demographics and attitude to those who did not.
- The data collection mode was different in Cuba than the other countries (paper for the former; electronic data collection for the latter), and the survey was only available to persons who worked at one of the institutions that was furnished with paper forms—all of which were in Havana.

### 4.4. Conclusions

HWs have a key role in promoting vaccine acceptance in their communities. Additionally, they are a key group for receiving vaccination themselves. Investing in the continued training of HWs on vaccination-related issues and interpersonal communication should be continuous so that they can detect and address misinformation more effectively and is essential to support vaccine acceptance. Moreover, activities programmed for the medium and long term should highlight professional staff training and evaluate whether the curricula are based on conceptual, procedural, and attitudinal competencies that enable HWs to carry out reflective and practical processes and to gain a comprehensive vision of vaccination. Additionally, social media and the influence of local, relevant leaders present an opportunity that may be particularly helpful in reaching specific subgroups of HWs. Specific communication campaigns targeted at HWs are needed. Messages and channels of communication should be tailored appropriately to these audiences.

## Data Availability

The survey instrument appears in the Supplementary Materials. A restricted set of de-identified survey response data will be made available on e-mail request to the corresponding author for a period of one year after publication of this article. Demographic variables and responses to Likert-type questions will be made available for all respondents.

## Supplementary Materials

The following supporting information can be downloaded at https://www.mdpi.com/article/10.3390/vaccines11091471/s1, File S1: Survey instrument (Spanish); File S2: Survey instrument (English); File S3: Percent of respondents, by demographic and COVID-19-related characteristics, region, and country of origin.
