# Supplementary material for: Understanding COVID-19 Vaccine Acceptance among Latin American Health Workers: Implications for Designing Interventions
Source: Vaccines (Basel). 2023 Sep 10;11(9):1471. doi: 10.3390/vaccines11091471 (PMC10536662; doi:10.3390/vaccines11091471)
Supplement: Supplementary file 1 [file vaccines-11-01471-s001.zip › File S1.pdf]

S1. Survey Instrument (Spanish)

Aceptación Vacunas contra COVID-19 en Trabajadores de la Salud América Latina

Start of Block: Default Question Block

Conocimientos, actitudes y prácticas de los trabajadores de salud sobre las vacunas contra la COVID-19 en América Latina

Muchas gracias por su interés en esta encuesta. El propósito del estudio es documentar los conocimientos, actitudes y prácticas de los trabajadores de la salud con respecto a las vacunas COVID-19 en los países de América Latina. Un/a trabajador/a de la salud es aquella persona que realiza actividades cuya finalidad fundamental es mejorar la salud en sus respectivas comunidades. Solo los trabajadores de la salud participarán en la encuesta. Estimamos que el cuestionario no tomará más de ocho (8) minutos para ser respondido.

Por favor lea el formulario de consentimiento y, si acepta participar, haga clic – SÍ - en el cuadro al final del formulario.

Q1 Confirmo que soy trabajador(a) de la salud

- ○ Si (1)
- ○ No (2)

Skip To: End of Survey If Confirmo que soy trabajador(a) de la salud = No

Consentimiento Informado
Título del Estudio: Conocimientos, Actitudes y Prácticas de los trabajadores de salud sobre las vacunas contra la COVID-19 en América Latina.
Investigadores: Organización Panamericana de la Salud (OPS) - Unidad de Inmunización Integral de la Familia - Departamento Familia, Promoción de la Salud y Curso de Vida: Tamara Rivera, Martha Velandia, Maite Vera, Dale Rhoda. Unidad de Sistemas y Servicios de Salud / Unidad de Recursos Humanos para la Salud - Departamento de Sistemas y Servicios de Salud: Silvia Cassiani, Malhi Cho, Hernan Sepúlveda.
Propósito: Documentar los conocimientos, actitudes y prácticas de los trabajadores de la salud con respecto a las vacunas contra la COVID-19 en los países de América Latina, lo que permitirá apoyar en el desarrollo y mejoramiento de estrategias y enfoques de comunicación sobre las vacunas contra la COVID-19 dirigidas a este grupo prioritario, con el objetivo de aumentar la aceptación de la vacuna y mejorar la confianza.
Potenciales Beneficios: El estudio apunta a una mejor comprensión de la aceptación de los trabajadores de la salud

hacia la vacuna contra la COVID-19, lo que permitirá a la OPS apoyar en el desarrollo y mejoramiento de estrategias de comunicación sobre las vacunas contra la COVID-19, y apoyar en el desarrollo de políticas públicas, para aumentar la aceptación de la vacuna, mejorando la confianza y, por lo tanto, permitiendo una promoción eficaz de la vacuna entre la población en general.

Potenciales Riesgos: No hay riesgos conocidos o esperados por participar en este estudio. La OPS compartirá los resultados con los ministerios de salud y las asociaciones profesionales utilizando información consolidada a nivel regional, por lo que no hay posibilidad de rastrear las respuestas hasta los participantes individuales.

Protección de Privacidad: Los investigadores que figuran en la primera página de este formulario son las únicas personas que tendrán acceso a la información que vincula a los participantes individuales con las respuestas de la encuesta.

Consentimiento

Entiendo que se me pide que participe en un estudio de la OPS/OMS para responder preguntas relacionadas con los conocimientos, actitudes y prácticas de los trabajadores de la salud con respecto a las vacunas contra la COVID-19 en América Latina. Entiendo que es mi decisión voluntaria de participar en este estudio y / o retirarme del estudio en cualquier momento. Se me proporcionará un resumen de los resultados de la encuesta al finalizar el estudio, en caso de que solicite una copia. Entiendo lo que implica este estudio y acepto participar libremente.

Si tiene alguna pregunta o inquietud, ya sea antes o después de su participación, no dude en comunicarse con:

Dra. Martha Velandia al +1 202-733-8269 o por email velandiam@paho.org
Dra. Tamara Rivera al +1 246- 851-1802 o por email riveratam@paho.org

Q2 País donde trabaja:

Argentina (1)
Bolivia 2
Colombia 3
Costa Rica 4
Cuba 5
Chile 6
Ecuador 7
El Salvador 8
Guatemala 9
Honduras 10
Nicaragua 11
Panamá 12
Paraguay 13
Perú 14
República Dominicana 15
Uruguay 16
Venezuela (17)

Q3 He leído la descripción del estudio y consiento participar en el mismo:

○ Si (1)

○ No (2)

Skip To: End of Survey If He leído la descripción del estudio y consiento participar en el mismo: = No

---

Q4 Sexo

○ Femenino (1)

○ Masculino (2)

○ Otro (3)

Q5 Edad (Años)

____

Q6 Etnia/Raza

- ◯ Afrodescendiente (1)
- ◯ Blanco (2)
- ◯ Indígena (3)
- ◯ Mestizo (4)
- ◯ Otros (5)

Display This Question:

If Etnia/Raza = Otros

Q6OPS Etnia/Raza - Otros: (*Por favor especificar)*

____

Q7 Sector donde trabaja *(Sector al cual le dedica más horas de trabajo. Si tiene varios trabajos con igual carga horaria, por favor escoja uno)*

- ◯ Público (1)
- ◯ Privado (2)
- ◯ Seguridad Social (3)
- ◯ Sector Académico (4)
- ◯ Otro (5)

Display This Question:

If Sector donde trabaja (Sector al cual le dedica más horas de trabajo. Si tiene varios trabajos con... = Otro

Q7OPS Sector donde trabaja - Otro: (*Por favor especificar*)

____

Q8 Lugar de trabajo *(De acuerdo con su respuesta anterior por favor seleccione el lugar donde dedica más horas de trabajo. Si tiene varios trabajos con igual carga horaria, por favor escoja uno)*

- ○ Servicios asistenciales de primer nivel de atención (centros de salud, consultorios privados, consultorios de empresas, consultorios de instituciones). (1)
- ○ Servicios asistenciales de otros niveles de atención (hospitales, clínicas, policlínicos, laboratorios). (2)
- ○ Instituciones del estado (Ministerios o Secretarías de Salud, Seguridad Social, entre otros). (3)
- ○ Instituciones educativas (escuelas, colegios, universidades, otros). (4)
- ○ Otros (5)

Display This Question:

If Lugar de trabajo (De acuerdo con su respuesta anterior por favor seleccione el lugar donde dedica... = Otros

Q8OPS Lugar de trabajo - Otros: *(Por favor especificar)*

____

Q9 Profesión

- ◯ Médico general (1)
- ◯ Médico especialista (clínico, quirúrgico y de urgencia) (2)
- ◯ Enfermero (licenciado) (3)
- ◯ Enfermero licenciado con especialidad (clínico, quirúrgico y de urgencia) (4)
- ◯ Profesional de partería (matrona/partera profesional/obstetriz) (5)
- ◯ Dentista/Odontólogo (6)
- ◯ Psicólogo (7)
- ◯ Farmacéutico (8)
- ◯ Profesional a cargo de la higiene y la salud medioambiental y ocupacional (9)
- ◯ Profesional de salud pública (10)
- ◯ Profesional de la gestión administrativa de salud (11)
- ◯ Fisioterapeuta (12)
- ◯ Dietista y nutricionista (13)
- ◯ Biólogo, Microbiólogo y bacteriólogo (14)
- ◯ Técnico de enfermería (15)
- ◯ Auxiliar de enfermería (16)
- ◯ Otro técnico en salud (17)
- ◯ Otro (18)

Display This Question:

If Profesión = Otro

Q9OPS Profesión - Otro: *(Por favor especificar)*

____

Q10 ¿Tiene, o ha tenido, la enfermedad de la COVID-19?:

- ○ Si (1)
- ○ No (2)
- ○ No sabe (3)

Q11 ¿Se ha realizado exámenes para saber si ha tenido, la enfermedad de la COVID 19?

- ○ Si (1)
- ○ No (2)
- ○ No sabe (3)

End of Block: Default Question Block

Start of Block: Block 5

Q12 **Por favor, comparta con nosotros cuánto está de acuerdo o en desacuerdo con las declaraciones a continuación.**

End of Block: Block 5

Start of Block: Block 1

X→

Q12 **Actitudes hacia las vacunas**

	Totalmente de acuerdo (1)	De acuerdo (2)	En desacuerdo (3)	Totalmente en desacuerdo (4)
Las vacunas son importantes para mi salud. (Q12a)	○	○	○	○
La vacunación es una buena manera de protegerme de las enfermedades. (Q12b)	○	○	○	○
En general, las vacunas son seguras. (Q12c)	○	○	○	○
En general, las vacunas son efectivas. (Q12d)	○	○	○	○
Vacunarse es importante para la salud de otros en mi comunidad. (Q12e)	○	○	○	○
La información que me dan las autoridades de Salud (Ministerio o Secretaría de Salud) es fiable y digna de confianza. (Q12f)	○	○	○	○
La información que me da mi prestador de atención de salud es fiable y digna de confianza. (Q12g)	○	○	○	○
En general, hago lo que mi médico o prestador de atención de salud recomienda con respecto a las vacunas para mí y mi familia. (Q12h)	○	○	○	○

Q13 **Predisposición con respecto a las vacunas**

	Totalmente de acuerdo (1)	De acuerdo (2)	En desacuerdo (3)	Totalmente en desacuerdo (4)
Las vacunas nuevas presentan más riesgos que las vacunas más antiguas. (Q13a)	○	○	○	○
Recomendaría una vacuna nueva a mis amigos y familiares. (Q13b)	○	○	○	○
Me preocupan los **efectos adversos leves** que las vacunas pueden causar en mí *(Son aquellos signos y síntomas fácilmente tolerados. No requieren terapia ni intervención médica)* (Q13c)	○	○	○	○
Me preocupan los **efectos adversos graves** que las vacunas pueden causar en mí *(Signos o síntomas que incapacitan e inhabilitan para efectuar actividades habituales)* (Q13d)	○	○	○	○

End of Block: Block 1

Start of Block: Block 2

X→

Q14 **Actitudes de los trabajadores de salud hacia las vacunas contra la COVID-19**

	Totalmente de acuerdo (1)	De acuerdo (2)	En desacuerdo (3)	Totalmente en desacuerdo (4)
La vacuna contra el coronavirus (COVID-19) me protegerá de las formas graves de la enfermedad de la COVID-19. (Q14a)	○	○	○	○
Confío en el proceso de aprobación científica de las vacunas disponibles contra el coronavirus (COVID-19) en mi país. (Q14b)	○	○	○	○
Vacunarme contra el coronavirus (COVID-19), es o será bueno para mi salud. (Q14c)	○	○	○	○
Recomendaría la vacuna contra la COVID-19 a **personas elegibles** *(Criterio establecido por el Ministerio de Salud de cada país. Puede incluir entre otros: empleados esenciales de primera línea, personas de la tercera edad, personas con una afección médica subyacente que aumenta su riesgo de enfermedad grave, mujeres embarazadas)* (Q14d)	○	○	○	○

Q15 **Disponibilidad de la vacuna contra el coronavirus (COVID-19):**

	Si (1)	No (2)
Conozco dónde acudir para ser vacunado/a. (Q15a)	○	○
Tengo acceso a los servicios de vacunación contra la COVID-19. (Q15b)	○	○

Display This Question:

If Disponibilidad de la vacuna contra el coronavirus (COVID-19): = Tengo acceso a los servicios de vacunación contra la COVID-19. [No]

Q16 Se me dificulta el acceso a la vacuna contra la COVID-19 debido a: *(Por favor, colocar comentario que responda una frase completa)*

____

Q17 **Disponibilidad de la vacuna contra el coronavirus (COVID-19):**

	Si (1)	No (2)
He recibido alguna vez una vacuna contra la COVID-19.	○	○

Display This Question:

If Disponibilidad de la vacuna contra el coronavirus (COVID-19): = He recibido alguna vez una vacuna contra la COVID-19. [Si]

X→

Q18 **Disponibilidad de la vacuna contra el coronavirus (COVID-19):**

	Si (1)	No (2)
He podido vacunarme contra la COVID-19 en **mi lugar de trabajo**	○	○

Display This Question:

If Disponibilidad de la vacuna contra el coronavirus (COVID-19): = He recibido alguna vez una vacuna contra la COVID-19. [Si]

X→

Q19 ¿Cuál de las siguientes dosis de la vacuna contra la COVID-19 ha recibido?:

- ◯ Dosis única (en esquema de una dosis) (1)
- ◯ Dosis única y dosis de refuerzo/dosis adicional (2)
- ◯ Primera dosis (en un esquema de dos dosis) (3)
- ◯ Primera y segunda dosis (en un esquema de dos dosis) (4)
- ◯ Primera dosis, segunda dosis y dosis de refuerzo/dosis adicional (5)
- ◯ Dos dosis de refuerzo/dosis adicional en cualquier esquema (de dosis única o de dos dosis) (6)

Display This Question:

If Disponibilidad de la vacuna contra el coronavirus (COVID-19): = He recibido alguna vez una vacuna contra la COVID-19. [No]

Q20 ¿Cuándo tiene la intención de vacunarse contra la COVID-19?

- ◯ Tan pronto, cuando sea elegible (1)
- ◯ No muy pronto, pero tal vez algún día (2)
- ◯ Nunca (3)

Display This Question:

If ¿Cuál de las siguientes dosis de la vacuna contra la COVID-19 ha recibido?: = Dosis única (en esquema de una dosis)

Or ¿Cuál de las siguientes dosis de la vacuna contra la COVID-19 ha recibido?: = Primera dosis (en un esquema de dos dosis)

Or ¿Cuál de las siguientes dosis de la vacuna contra la COVID-19 ha recibido?: = Primera y segunda dosis (en un esquema de dos dosis)

Q21 No cuento con el esquema completo de vacunación o con las dosis de refuerzo/dosis adicional contra la enfermedad COVID-19 debido a: *(Por favor, responda con una frase completa sus razones)*

____

Display This Question:

If ¿Cuál de las siguientes dosis de la vacuna contra la COVID-19 ha recibido?: = Primera dosis (en un esquema de dos dosis)

Or ¿Cuál de las siguientes dosis de la vacuna contra la COVID-19 ha recibido?: = Dosis única (en esquema de una dosis)

Or ¿Cuál de las siguientes dosis de la vacuna contra la COVID-19 ha recibido?: = Primera y segunda dosis (en un esquema de dos dosis)

Q22 ¿Cuándo tiene la intención de recibir la próxima dosis de la vacuna COVID-19?

- ○ Tan pronto, cuando sea elegible (1)
- ○ No muy pronto, pero tal vez algún día (2)
- ○ Nunca (3)

Display This Question:

If Disponibilidad de la vacuna contra el coronavirus (COVID-19): = He recibido alguna vez una vacuna contra la COVID-19. [No]

Or ¿Cuál de las siguientes dosis de la vacuna contra la COVID-19 ha recibido?: = Dosis única (en esquema de una dosis)

Or ¿Cuál de las siguientes dosis de la vacuna contra la COVID-19 ha recibido?: = Primera dosis (en un esquema de dos dosis)

Or ¿Cuál de las siguientes dosis de la vacuna contra la COVID-19 ha recibido?: = Primera y segunda dosis (en un esquema de dos dosis)

X→

Q23 Por favor indique sus motivos para retrasar o rechazar las vacunas contra la COVID-19

	Totalmente de acuerdo (1)	De acuerdo (2)	En desacuerdo (3)	Totalmente en desacuerdo (4)
Todavía no sé lo suficiente sobre las vacunas disponibles para tomar una decisión. (Q23a)	○	○	○	○
Quiero obtener inmunidad natural al virus que causa la COVID-19. (Q23b)	○	○	○	○
Es posible que las vacunas disponibles se hayan desarrollado demasiado rápido o que no se la hayan sometido a pruebas exhaustivas antes de su aprobación. (Q23c)	○	○	○	○
Creo que las vacunas pueden causar la enfermedad que fueron diseñadas para prevenir. (Q23d)	○	○	○	○
Me preocupan las reacciones adversas que se han visto cuándo se colocan la vacuna. (Q23e)	○	○	○	○

Display This Question:

If Disponibilidad de la vacuna contra el coronavirus (COVID-19): = He recibido alguna vez una vacuna contra la COVID-19. [No]

Or ¿Cuál de las siguientes dosis de la vacuna contra la COVID-19 ha recibido?: = Dosis única (en esquema de una dosis)

Or ¿Cuál de las siguientes dosis de la vacuna contra la COVID-19 ha recibido?: = Primera dosis (en un esquema de dos dosis)

Or ¿Cuál de las siguientes dosis de la vacuna contra la COVID-19 ha recibido?: = Primera y segunda dosis (en un esquema de dos dosis)

Q24 Otras razones por retrasar o rechazar la vacuna contra la COVID-19: *(Por favor, responda con una frase completa sus razones)*

____

Q25 Los siguientes factores contribuyeron a formar mi opinión sobre las vacunas contra la COVID-19:

	Totalmente de acuerdo (1)	De acuerdo (2)	En desacuerdo (3)	Totalmente en desacuerdo (4)
La rapidez con la que se investigaron y desarrollaron las vacunas. (Q25a)	○	○	○	○
Los temas científicos del SARS-CoV-2 que se descubren y evolucionan constantemente. (Q25b)	○	○	○	○
Las recomendaciones que emiten los científicos y organismos internacionales. (Q25c)	○	○	○	○
Las acciones y opiniones de mis amigos, familiares y colegas sobre las vacunas. (Q25d)	○	○	○	○
Las acciones y opiniones de mis líderes religiosos. (Q25e)	○	○	○	○
La relación entre la proporción de vacunados con hospitalización y mortalidad. (Q25f)	○	○	○	○
Mi propia investigación sobre las vacunas contra la COVID-19. (Q25g)	○	○	○	○
El país en el que se desarrollaron/fabricaron las vacunas disponibles. (Q25h)	○	○	○	○
La información que he visto en las redes sociales (Facebook, Instagram, Twitter, Tiktok, Youtube, WhatsApp, otros). (Q25i)	○	○	○	○

Q26 Otros factores que contribuyeron a formar mi opinión sobre las vacunas contra la COVID-19 *(por favor describa/especifique en el espacio correspondiente)*

____

End of Block: Block 2

Start of Block: Block 3

X→

Q27 Actitudes de los trabajadores de salud hacia la vacuna contra la **influenza**

	Totalmente de acuerdo (1)	De acuerdo (2)	En desacuerdo (3)	Totalmente en desacuerdo (4)
Recibiría la **vacuna contra la influenza** si me la ofrecieran.	○	○	○	○

Display This Question:

If Actitudes de los trabajadores de salud hacia la vacuna contra la influenza = Recibiría la vacuna contra la influenza si me la ofrecieran. [En desacuerdo]

Or Actitudes de los trabajadores de salud hacia la vacuna contra la influenza = Recibiría la vacuna contra la influenza si me la ofrecieran. [Totalmente en desacuerdo]

Q28 Si no está de acuerdo, ¿cuáles son las razones? *(Por favor, responda con una frase completa sus razones)*

____

____

____

____

____

X→

Q29 Actitudes de los trabajadores de salud hacia la vacuna contra la **influenza**

	Totalmente de acuerdo (1)	De acuerdo (2)	En desacuerdo (3)	Totalmente en desacuerdo (4)
Recomendaría la **vacuna contra la influenza** a mis amigos y familiares.	○	○	○	○

Display This Question:

If Actitudes de los trabajadores de salud hacia la vacuna contra la influenza = Recomendaría la vacuna contra la influenza a mis amigos y familiares. [En desacuerdo]

Or Actitudes de los trabajadores de salud hacia la vacuna contra la influenza = Recomendaría la vacuna contra la influenza a mis amigos y familiares. [Totalmente en desacuerdo]

Q30 Si no está de acuerdo, ¿cuáles son las razones? *(Por favor, responda con una frase completa sus razones)*

____

____

____

____

____

Q31 **Actitudes de los trabajadores de salud hacia la vacuna contra la hepatitis B**

	Totalmente de acuerdo (1)	De acuerdo (2)	En desacuerdo (3)	Totalmente en desacuerdo (4)
Recibiría la **vacuna contra la hepatitis B** si me la ofrecieran.	○	○	○	○

Display This Question:

If Actitudes de los trabajadores de salud hacia la vacuna contra la hepatitis B = Recibiría la vacuna contra la hepatitis B si me la ofrecieran. [En desacuerdo]

Or Actitudes de los trabajadores de salud hacia la vacuna contra la hepatitis B = Recibiría la vacuna contra la hepatitis B si me la ofrecieran. [Totalmente en desacuerdo]

Q32 Si no está de acuerdo, ¿cuáles son las razones? (Por favor, responda con una frase completa sus razones)

____

____

____

____

____

Q33 **Actitudes de los trabajadores de salud hacia la vacuna contra la hepatitis B**

	Totalmente de acuerdo (1)	De acuerdo (2)	En desacuerdo (3)	Totalmente en desacuerdo (4)
Recomendaría la **vacuna contra la hepatitis B** a mis colegas.	◯	◯	◯	◯

Display This Question:

If Actitudes de los trabajadores de salud hacia la vacuna contra la hepatitis B = Recomendaría la vacuna contra la hepatitis B a mis colegas. [En desacuerdo]

Or Actitudes de los trabajadores de salud hacia la vacuna contra la hepatitis B = Recomendaría la vacuna contra la hepatitis B a mis colegas. [Totalmente en desacuerdo]

Q34 Si no está de acuerdo, ¿cuáles son las razones? *(Por favor, responda con una frase completa sus razones)*

____

____

____

____

____

End of Block: Block 3

Start of Block: Block 4

Display This Question:

If He leído la descripción del estudio y consiento participar en el mismo: = No

Gracias por considerar realizar esta encuesta. Que tenga un buen día.

Skip To: End of Block If Gracias por considerar realizar esta encuesta. Que tenga un buen día. Is Displayed

End of Block: Block 4

Start of Block: Block 6

Q35 Si existe algún comentario adicional acerca de las vacunas contra la COVID-19 para los trabajadores de la salud, por favor escríbalo aquí:

____

____

____

____

____

End of Block: Block 6
