# Supplementary material for: Understanding COVID-19 Vaccine Acceptance among Latin American Health Workers: Implications for Designing Interventions
Source: Vaccines (Basel). 2023 Sep 10;11(9):1471. doi: 10.3390/vaccines11091471 (PMC10536662; doi:10.3390/vaccines11091471)
Supplement: Supplementary file 1 [file vaccines-11-01471-s001.zip › File S2.pdf]

S2. Survey Instrument (English)

Acceptance of COVID-19 Vaccines in Latin American Health Workers

Start of Block: Default Question Block

Knowledge, attitudes, and practices of health workers about COVID-19 vaccines in Latin America

Thank you very much for your interest in this survey. The purpose of the study is to document the knowledge, attitudes, and practices of health workers regarding COVID-19 vaccines in Latin American countries. A health worker is a person who carries out activities whose fundamental purpose is to improve health in their respective communities. Only health workers will participate in the survey. We estimate that the questionnaire will take no more than eight (8) minutes to complete. Please read the consent form and, if you agree to participate, click – YES – in the box at the bottom of the form.

Q1 I confirm that I am a health worker

- ○ Yes (1)
- ○ No (2)

skip To : End of Survey If I confirm that I am a health worker = No

Informed Consent

Study Title: Knowledge, Attitudes and Practices of health workers about COVID-19 vaccines in Latin America.

Researchers: Pan American Health Organization (PAHO) - Comprehensive Family Immunization Unit - Family, Health Promotion and Life Course Department: Tamara Rivera, Martha Velandia, Maite Vera, Dale Rhoda. Health Systems and Services Unit / Human Resources for Health Unit - Department of Health Systems and Services: Silvia Cassiani, Malhi Cho, Hernan Sepúlveda.

Purpose: Document the knowledge, attitudes and practices of health workers regarding vaccines against COVID-19 in Latin American countries, which will allow support in the development and improvement of communication strategies and approaches on vaccines. against COVID-19 aimed at this priority group, with the aim of increasing the acceptance of the vaccine and improving confidence.

Potential Benefits: The study points to a better understanding of the acceptance of health workers towards the COVID-19 vaccine, which will allow PAHO to support the development and improvement of communication strategies on COVID vaccines. -19, and support the development of public policies, to increase the acceptance of the vaccine, improving confidence and, therefore, allowing an effective promotion of the vaccine among the general population.

Potential Risks: There are no known or expected risks from participating in this study. PAHO will share the results with ministries of health and professional associations using information consolidated at the regional level, so there is no possibility of tracing responses back to individual participants.

Privacy Protection: The researchers listed on the first page of this form are the only people who will have access to information linking individual participants to survey responses.

Consent

I understand that I am being asked to participate in a PAHO/WHO study to answer questions related to health workers' knowledge, attitudes, and practices regarding COVID-19 vaccines in Latin America. I understand that it is my voluntary decision to participate in this study and/or withdraw from the study at any time. A summary of the survey results will be provided to me at the end of the study, should I request a copy. I understand what this study involves and I freely agree to participate. If you have any questions or concerns, either before or after your participation, please do not hesitate to contact:

Dr. Martha Velandia at +1 202-733-8269 or email velandiam@paho.org

Dr. Tamara Rivera at +1 246 - 851-1802 or by email riveratam@paho.org

Q2 Country where you work:

Argentinia 1
Bolivia 2
Colombia 3
Costa Rica 4
Cuba 5
Chile 6
Ecuador 7
El Salvador 8
Guatemala 9
Honduras 10
Nicaragua 11
Panama 12
Paraguay 13
Peru 14
Dominican Republic 15
Uruguay 16
Venezuela 17

Q3 I have read the description of the study and I consent to participate in it:

- Yes (1)
- No (2)

skip To : End of Survey If I have read the description of the study and I agree to participate in it: = No

Q4 Gender

- Female (1)
- Male (2)
- Other (3)

Q5 Age (Years)

____

Q6 Ethnicity/Race

- ○ Afro-descendant (1)
- ○ White (2)
- ○ Indigenous (3)
- ○ Mixed race (4)
- ○ Others (5)

Display This Question:

If Ethnicity /Race = Others

Q6OPS Ethnicity/Race - Other: (*Please specify)*

____

Q7 Sector where you work *(Sector to which you dedicate more hours of work. If you have several jobs with the same workload, please choose one)*

- ○ Public (1)
- ○ Private (2)
- ○ Security Community (3)
- ○ Academic Sector (4)
- ○ Other (5)

Display This Question:

If Sector where you work (Sector to which you spend more hours of work. If you have various jobs with... = Other

Q7OPS Sector where you work - Other: (*Please specify*)

____

Q8 Place of work *(According to your previous answer, please select the place where you spend more hours of work. If you have several jobs with the same workload, please choose one)*

- ◯ First-level care services (health centers, private clinics, company clinics, institutional clinics). (one)
- ◯ Care services at other levels of care (hospitals, clinics, polyclinics, laboratories). (two)
- ◯ State institutions (Ministries or Secretaries of Health, Social Security, among others). (3)
- ◯ Educational institutions (schools, colleges, universities, others). (4)
- ◯ Others (5)

Display This Question:

If Workplace (According to your previous answer, please select the place where you work... = Others

Q8OPS Workplace - Other: *(Please specify)*

____

Q9 Profession

- ○ Doctor overall (1)
- ○ Medical specialist (clinical, surgical and emergency) (2)
- ○ Nurse (graduate) (3)
- ○ Licensed nurse with specialty (clinical, surgical and emergency) (4)
- ○ Midwifery professional (midwife/professional midwife/ obstetrician) (5)
- ○ Dentist / Dentist (6)
- ○ Psychologist (7)
- ○ Pharmacist (8)
- ○ Professional in charge of hygiene and environmental and occupational health (9)
- ○ Public health professional (10)
- ○ Health administrative management professional (11)
- ○ Physiotherapist (12)
- ○ Dietician and nutritionist (13)
- ○ Biologist , Microbiologist and Bacteriologist (14)
- ○ Nursing Technician (15)
- ○ Nursing assistant (16)
- ○ Other technical in health (17)
- ○ Other (18)

Display This Question:

If Profession = Other

Q9OPS Profession - Other: *(Please specify)*

____

Q10 Do you have, or have you had, the COVID-19 disease?:

- Yes (1)
- No (2)
- Don't know (3)

Q11 Have you had tests to find out if you have had the COVID 19 disease?

- Yes (1)
- No (2)
- Don't know (3)

End of Block: Default Question Block

Please share with us how much you agree or disagree with the statements below.

Q12 **Attitudes towards vaccines**

	Completely Agree (1)	Agree (2)	Disagree (3)	Completely disagree (4)
Vaccines are important to my health. (Q12a)	○	○	○	○
Vaccination is a good way to protect myself from diseases. (Q12b)	○	○	○	○
In general, vaccines are safe. (Q12c)	○	○	○	○
In general, vaccines are effective. (Q12d)	○	○	○	○
Getting vaccinated is important to the health of others in my community. (Q12e)	○	○	○	○
The information given to me by the Health authorities (Ministry or Secretary of Health) is reliable and trustworthy. (Q12f)	○	○	○	○
The information my health care provider gives me is reliable and trustworthy. (Q12g)	○	○	○	○
In general, I do what my doctor or health care provider recommends regarding vaccinations for me and my family. (Q12h)	○	○	○	○

Q13 **Predisposition regarding vaccinations**

	Completely Agree (1)	Agree (2)	Disagree (3)	Completely disagree (4)
Newer vaccines pose more risks than older vaccines. (Q13a)	○	○	○	○
I would recommend a new vaccine to my friends and family. (Q13b)	○	○	○	○
I am concerned about the **minor adverse effects** that the vaccines may cause in me *(these are those signs and symptoms that are easily tolerated. They do not require therapy neither intervention medical)* (Q13c)	○	○	○	○
I am concerned about the **serious adverse effects** that vaccines can cause in me *(Signs or symptoms that incapacitate and disable me from carrying out usual activities)* (Q13d)	○	○	○	○

End of Block: Block 1

Start of Block: Block 2

Q14 **Attitudes of health workers towards COVID-19 vaccines**

	Completely Agree (1)	Agree (2)	Disagree (3)	Completely disagree (4)
The coronavirus (COVID-19) vaccine will protect me from severe forms of the COVID-19 disease. (Q14a)	○	○	○	○
I trust the scientific approval process of the available vaccines against coronavirus (COVID-19) in my country. (Q14b)	○	○	○	○
Getting vaccinated against the coronavirus (COVID-19), is or will be good for my health. (Q14c)	○	○	○	○
I would recommend the COVID-19 vaccine to **eligible people** *(Criteria established by the Ministry of Health of each country. May include but are not limited to: essential front-line employees, the elderly, people with an underlying medical condition that increases their risk of serious illness, pregnant women)* (Q14d)	○	○	○	○

Q15 **Availability of the coronavirus vaccine (COVID-19):**

	Yes (1)	No (2)
I know where to go to be vaccinated. (Q15a)	○	○
I have access to COVID-19 vaccination services. (Q15b)	○	○

Display This Question:

If Coronavirus (COVID-19) Vaccine Availability: = I have access to COVID-19 vaccination services. [No]

Q16 It is difficult for me to access the COVID-19 vaccine due to:
(Please post a comment that answers a complete sentence)

____

Q17 **Availability of the coronavirus vaccine (COVID-19):**

	Yes (1)	No (2)
I have ever received a vaccine against COVID-19.	○	○

Display This Question:

If Coronavirus (COVID-19) Vaccine Availability: = I have ever received a COVID-19 vaccine. [Yes]

Q18 **Availability of the coronavirus vaccine (COVID-19):**

	Yes (1)	No (2)
I have been able to get vaccinated against COVID-19 at **my workplace**	○	○

Display this Question :

If Coronavirus (COVID-19) Vaccine Availability: = I have ever received a COVID-19 vaccine. [Yes]

Q19 Which of the following doses of the COVID-19 vaccine have you received?:

- ○ Single dose (on a one- dose schedule) (1)
- ○ Single dose and booster dose/ additional dose (2)
- ○ First dose (in a two- dose schedule) (3)
- ○ First and second dose (in a two- dose schedule) (4)
- ○ First dose, second dose and booster dose/ additional dose (5)
- ○ Two booster doses/additional dose in any schedule (single dose or two doses) (6)

Display this Question:

If Coronavirus (COVID-19) Vaccine Availability: = I have ever received a COVID-19 vaccine. [No]

Q20 When do you intend to get vaccinated against COVID-19?

- ○ As soon as eligible (1)
- ○ Not very soon, but maybe someday (2)
- ○ Never (3)

Display This Question:

If Which of the following doses of the COVID-19 vaccine have you received?: = Single dose (on a one-dose schedule)

Or Which of the following doses of the COVID-19 vaccine have you received: = First dose (in a two-dose schedule)

Or Which of the following doses of the COVID-19 vaccine have you received: = First and second doses (in a two-dose schedule)

Q21 I do not have the complete vaccination schedule or the booster doses/additional doses against the COVID-19 disease due to: *(Please answer your reasons in a complete sentence)*

____

Display this Question:

If Which of the following doses of the COVID-19 vaccine have you received: = First dose (in a two-dose schedule)

Or Which of the following doses of the COVID-19 vaccine have you received: = Single dose (on a one-dose schedule)

Or Which of the following doses of the COVID-19 vaccine have you received: = First and second doses (in a two-dose schedule)

Q22 When do you intend to receive the next dose of the COVID-19 vaccine?

- ○ As soon as eligible (1)
- ○ Not very soon, but maybe someday (2)
- ○ Never (3)

Display This Question:

If Coronavirus (COVID-19) Vaccine Availability: = I have ever received a COVID-19 vaccine. [No]

Or Which of the following doses of the COVID-19 vaccine have you received: = Single dose (on a one-dose schedule)

Or Which of the following doses of the COVID-19 vaccine have you received: = First dose (in a two-dose schedule)

Or Which of the following doses of the COVID-19 vaccine have you received: = First and second doses (in a two-dose schedule)

Q23 Please indicate your reasons for delaying or refusing COVID-19 vaccines

	Completely Agree (1)	Agree (2)	Disagree (3)	Completely disagree (4)
I still don't know enough about the available vaccines to make a decision. (Q23a)	◯	◯	◯	◯
I want to gain natural immunity to the virus that causes COVID-19. (Q23b)	◯	◯	◯	◯
Available vaccines may have been developed too quickly or not thoroughly tested before approval. (Q23c)	◯	◯	◯	◯
I believe that vaccines can cause the disease they were designed to prevent. (Q23d)	◯	◯	◯	◯
I am concerned about the adverse reactions that have been seen when the vaccine is given. (Q23e)	◯	◯	◯	◯

Display This Question:

If Coronavirus (COVID-19) Vaccine Availability: = I have ever received a COVID-19 vaccine. [No]

Or Which of the following doses of the COVID-19 vaccine have you received: = Single dose (on a one-dose schedule)

Or Which of the following doses of the COVID-19 vaccine have you received: = First dose (in a two-dose schedule)

Or Which of the following doses of the COVID-19 vaccine have you received: = First and second doses (in a two-dose schedule)

Q24 Other reasons for delaying or refusing the COVID-19 vaccine:
(Please answer your reasons in a complete sentence)

____

Q25 The following factors contributed to my opinion of COVID-19 vaccines:

	Completely Agree (1)	Agree (2)	Disagree (3)	Completely disagree (4)
The speed with which vaccines were researched and developed. (Q25a)	○	○	○	○
The scientific topics of SARS-CoV-2 that are constantly being discovered and evolving. (Q25b)	○	○	○	○
The recommendations issued by scientists and international organizations. (Q25c)	○	○	○	○
The actions and opinions of my friends, family, and colleagues about vaccines. (Q25d)	○	○	○	○
The actions and opinions of my religious leaders. (Q25e)	○	○	○	○
The relationship between the proportion of vaccinated with hospitalization and mortality. (Q25f)	○	○	○	○
My own research on COVID-19 vaccines. (Q25g)	○	○	○	○
The country in which the available vaccines were developed/manufactured. (Q25h)	○	○	○	○
The information I have seen on social networks (Facebook, Instagram, Twitter, Tiktok , Youtube , WhatsApp, others). (Q25i)	○	○	○	○

Q26 Other factors that contributed to my opinion about COVID-19 vaccines
(please describe/specify in the space provided)

____

End of Block: Block 2

Start of Block: Block 3

Q27 Attitudes of health workers towards the **influenza vaccine**

	Completely Agree (1)	Agree (2)	Disagree (3)	Completely disagree (4)
I would get the **flu shot** if it was offered to me.	○	○	○	○

Display This Question:

If Health worker attitudes towards influenza vaccination = I would receive the < strong >influenza vaccine</ strong > if it was offered to me. [Disagree]

Or Attitudes of health workers towards influenza vaccination = I would receive the < strong >influenza vaccine</ strong > if it was offered to me. [Strongly disagree]

Q28 If you do not agree, what are the reasons? *(Please, answer with a complete sentence your reasons)*

____

Q29 Attitudes of health workers towards the **influenza vaccine**

	Completely Agree (1)	Agree (2)	Disagree (3)	Completely disagree (4)
I would recommend the **flu vaccine** to my friends and family.	○	○	○	○

Display This Question:

If Health care workers' attitudes toward influenza vaccination = I would recommend the < strong >influenza vaccine</ strong > to my friends and family. [Disagree]

Or Attitudes of health care workers towards influenza vaccination = I would recommend the < strong >influenza vaccine</ strong > to my friends and family. [Strongly disagree]

Q30 If you do not agree, what are the reasons? *(Please, answer with a complete sentence your reasons)*

____

Q31 **Attitudes of health workers towards the hepatitis B vaccine**

	Completely Agree (1)	Agree (2)	Disagree (3)	Completely disagree (4)
I would get the **hepatitis B vaccine** if it was offered to me.	○	○	○	○

Display This Question:

If Attitudes of health workers towards the hepatitis B vaccine = I would receive the < strong >hepatitis B vaccine </ strong >if it was offered to me. [Disagree]

Or Attitudes of health workers towards the hepatitis B vaccine = I would receive the < strong >hepatitis B vaccine </ strong >if it was offered to me. [Strongly disagree]

Q32 If you do not agree, what are the reasons? (Please, answer with a complete sentence your reasons)

____

Q33 **Attitudes of health workers towards the hepatitis B vaccine**

	Completely Agree (1)	Agree (2)	Disagree (3)	Completely disagree (4)
I would recommend the **hepatitis B vaccine** to my colleagues.	○	○	○	○

Display This Question:

If Attitudes of health workers towards the hepatitis B vaccine = I would recommend the < strong >hepatitis B vaccine</ strong > to my colleagues. [Disagree]

Or Attitudes of health workers towards the hepatitis B vaccine = I would recommend the < strong >hepatitis B vaccine</ strong > to my colleagues. [Strongly disagree]

Q34 If you do not agree, what are the reasons? *(Please, answer with a complete sentence your reasons)*

____

End of Block: Block 3

Start of Block: Block 4

Display this Question:

If I have read the description of the study and I agree to participate in it: = No

Thank you for considering taking this survey. Have a nice day.

skip To : End of Block If Thank you for considering taking this survey. Have a nice day. Is Displayed

End of Block: Block 4

Start of Block: Block 6

Q35 If there are any additional comments about COVID-19 vaccines for healthcare workers, please write them here:

____

End of Block: Block 6
