# Supplementary material for: Understanding COVID-19 Vaccine Acceptance among Latin American Health Workers: Implications for Designing Interventions
Source: Vaccines (Basel). 2023 Sep 10;11(9):1471. doi: 10.3390/vaccines11091471 (PMC10536662; doi:10.3390/vaccines11091471)
Supplement: Supplementary file 1 [file vaccines-11-01471-s001.zip › File S3.pdf]

S3. Percent of respondents, by demographic and COVID-19-related characteristics, region, and country of origin

Respondent Demographic and COVID-19-related Characteristics	Respondent Region and Country of Origin																			
	Central America								Andean Community						Southern Cone					All 16 Countries
	Costa Rica	Cuba	Dominican Republic	El Salvador	Guatemala	Honduras	Panama	Total	Bolivia	Colombia	Ecuador	Peru	Venezuela	Total	Argentina	Chile	Paraguay	Uruguay	Total	
**All**																				
N (unweighted)	1038	307	207	264	318	414	307	2855	293	1056	517	219	305	2390	324	458	259	432	1473	6718
N (Weighted by number of doctors and nurses in the country)	36,179	181,198	31,264	30,205	62,293	11,939	20,544	373,622	28,977	192,488	80,074	121,853	110,556	533,948	297,694	121,716	17,899	41,405	478,714	1,386,284
**Healthcare Worker Type**																				
Physicians	22	24	51	46	42	31	46	32	23	11	34	29	32	24	41	52	34	47	44	33
Licensed Nurses and Midwives	20	31	5	25	11	17	3	22	45	60	31	44	26	44	22	23	31	12	22	30
Other Health Professionals	25	30	36	14	11	29	45	26	11	23	25	16	26	22	21	18	22	13	19	22
Health Technicians	20	4	3	11	28	14	3	10	19	2	5	8	8	6	7	4	2	21	7	8
Others	13	10	5	5	9	9	4	9	2	5	5	3	9	5	9	4	10	6	8	7
**Gender**																				
Male	28	27	21	31	42	27	38	30	29	20	33	34	18	25	23	36	22	19	26	27
Female	72	72	79	69	58	73	63	70	71	80	67	66	82	75	77	64	78	82	74	73
Other	0	0	0	0	0	0	0	0	0	0	0	0	0	0	0	0	0	0	0	0
**Age**	Q1 (19-33) - Q2 (34-43) - Q3 (44-52) - Q4 (53-82)								Q1 (20-31) - Q2 (32-40) - Q3 (41-51) - Q4 (52-81)						Q1 (22-40) - Q2 (41-51) - Q3 (52-59) - Q4 (60-83)					Q1 (19-34) - Q2 (35-44) - Q3 (45-55) - Q4 (56-83)
Age quartile 1	27	13	41	41	60	39	8	28	18	41	37	9	9	25	19	39	44	35	26	26
Age quartile 2	38	16	34	27	24	26	29	23	42	26	32	22	31	28	26	20	30	34	25	26
Age quartile 3	19	35	12	20	10	17	22	25	25	18	22	28	28	23	28	15	18	19	24	24
Age quartile 4	16	36	14	13	6	18	41	25	14	15	9	42	33	24	27	26	8	12	25	25
**Race/Ethnicity**																				
Afro-descendant	2	14	7	0	0	1	14	8	0	6	16	1	6	6	0	0	0	1	0	5
White	56	53	12	19	5	9	27	36	6	28	3	4	35	19	83	59	55	94	77	44
Indigenous	1	0	1	2	11	1	2	2	9	2	1	2	0	2	0	1	0	0	1	2
Mixed race	38	32	77	75	79	89	52	51	81	56	78	94	56	69	13	37	42	4	20	47
Others	3	0	3	4	6	1	5	2	4	8	2	0	3	4	3	2	3	1	3	3
**Work Sector**																				
Public	62	98	69	69	92	73	55	85	93	34	86	81	83	66	62	72	77	23	62	70
Private	8	0	24	16	3	15	8	5	2	55	10	6	9	25	30	19	11	73	31	22
Social Security	30	0	1	10	3	1	35	6	3	1	1	11	1	3	2	0	5	1	2	4
Academic Sector	1	2	5	4	3	8	1	2	1	6	2	1	2	3	5	7	6	3	5	4
Other	0	1	2	1	0	2	1	1	1	3	1	1	5	3	1	1	2	1	1	2
**Workplace**																				
First Level Care Services (Health Centers, Private Offices, Business Offices, Institutional Offices)	45	27	18	35	13	32	15	26	61	23	63	35	38	37	30	45	24	23	33	33
Assistance Services From Other Levels Of Care (Hospitals, Clinics, Polyclinics, Laboratories)	30	43	42	33	71	30	56	46	18	45	16	37	21	32	48	39	32	67	47	41
State Institutions (Ministries Or Health Secretariats, Social Security, Among Others)	21	22	31	23	13	25	26	21	17	13	14	21	32	19	12	6	31	2	11	17
Educational Institutions (Schools, Colleges, Universities, Others)	1	3	5	5	1	9	2	3	1	10	4	1	3	5	5	7	7	4	6	5
Others	3	5	4	4	3	5	2	4	3	9	3	6	7	7	5	3	7	3	4	5
**Had COVID-19**																				
Yes	40	44	63	59	51	60	39	48	74	47	63	63	60	57	48	34	65	42	44	50
No	54	54	33	32	47	35	60	49	24	47	30	34	36	38	48	65	32	56	52	46
Do not know	6	2	3	9	2	5	2	3	3	6	7	3	3	5	4	2	3	2	3	4
**Tested for COVID-19**																				
Yes	76	92	94	89	88	95	95	90	93	88	90	94	85	89	77	91	92	91	82	87
No	24	9	6	11	12	5	5	10	7	12	10	6	15	11	23	9	8	10	17	13
Do not know	0	0	0	0	0	0	0	0	0	0	0	0	0	0	1	0	0	0	0	0
**Know where to go to be vaccinated for COVID-19**																				
Yes	100	100	100	100	98	100	99	99	99	99	100	99	100	99	100	99	100	99	100	99
No	0	0	1	0	2	0	1	1	1	1	0	1	0	1	0	1	0	1	0	1
**Have access to COVID-19 vaccination services**																				
Yes	100	97	99	99	98	100	99	98	100	99	99	99	99	99	99	100	100	100	100	99
No	0	3	1	1	2	0	1	2	0	1	1	1	1	1	1	0	0	0	0	1
**Ever been vaccinated for COVID-19**																				
Yes	99	100	99	99	99	100	100	100	100	99	99	100	99	99	99	99	99	100	99	99
Single dose (on a one- dose schedule)	0	2	1	2	1	0	0	1	1	1	1	1	1	1	0	0	0	0	0	1
Single dose and booster dose/ additional dose	3	9	7	3	6	5	2	7	6	5	5	3	4	5	3	2	3	2	2	4
First dose (in a two- dose schedule)	0	0	3	2	1	1	0	1	1	1	2	1	4	2	0	0	0	0	0	1
First and second dose (in a two-dose schedule)	3	3	25	16	7	5	4	7	15	12	9	2	18	11	4	1	4	4	3	7
First dose, second dose and booster dose/ additional dose	87	81	56	67	74	82	89	77	64	74	74	83	70	75	86	28	83	77	71	74
Two booster doses/additional dose in any schedule (single dose or two doses)	6	5	7	9	10	7	5	7	12	6	8	11	2	7	6	68	9	16	23	12
No Response (for the doses received)	0.0000	0.0000	0.0000	0.0000	0.0000	0.0000	0.0000	0.0000	0.0000	0.0000	0.0000	0.0000	0.0000	0.0000	0	0	0	0	0	0
No	1	0	1	1	1	0	0	1	0	1	2	1	1	1	1	2	1	1	1	1
**Able to be vaccinated at workplace**																				
Yes	90	95	84	70	81	82	90	89	89	74	89	81	90	82	65	82	77	44	68	79
No	5	1	9	21	12	13	3	6	2	19	5	10	3	11	28	10	13	48	24	14
No Response	5	4	6	8	6	5	8	5	9	6	4	9	6	7	7	6	9	8	7	6
Skipped due to previous response	1	0	1	1	1	0	0	1	0	1	2	1	1	1	1	2	1	1	1	1
**When will receive first vaccine dose (among those not yet vaccinated)**																				
As soon as you are eligible	0	0	0	0	0	0	0	0	0	0	1	0	1	0	0	0	0	0	0	0
Not very soon, but maybe one day	0	0	1	1	0	0	0	0	0	1	0	1	0	0	1	1	0	1	1	0
Never	1	0	1	0	0	0	0	0	0	0	0	0	0	0	0	1	0	0	0	0
Skipped due to previous response	99	100	99	99	99	100	100	100	100	99	99	100	99	99	99	99	99	100	99	99
**When intend to receive next vaccine dose (among those partially vaccinated)**																				
As soon as you are eligible	2	5	9	11	9	5	2	6	13	10	9	1	20	10	3	0	5	2	2	6

Not very soon, but maybe one day	1	0	15	7	1	0	1	2	3	4	2	1	2	3	2	0	0	1	1	2
Never	1	0	4	1	0	1	1	1	1	0	1	1	0	0	0	1	0	1	0	0
No Response	1	0	1	1	1	0	0	1	0	1	2	1	1	1	1	2	1	1	1	1
Skipped due to previous response	96	95	71	80	90	94	96	91	83	85	87	97	76	86	95	97	95	95	96	91

Cells hold the weighted column % of respondents.

Figures in each column sum to 100% in each section of the table.

Ever been vaccinated for COVID-19 section: The YES and NO rows will sum up to 100%. The Vaccination details will sum up to the YES %.
