# Supplementary material for: Understanding COVID-19 Vaccine Acceptance among Latin American Health Workers: Implications for Designing Interventions
Source: Vaccines (Basel). 2023 Sep 10;11(9):1471. doi: 10.3390/vaccines11091471 (PMC10536662; doi:10.3390/vaccines11091471)
Supplement: Supplementary file 1 [file vaccines-11-01471-s001.zip › Table of Contents.pdf]

Supplementary Materials

Understanding COVID-19 Vaccine Acceptance among Latin American Health Workers: Implications for Designing Interventions

Rivera, T.; Brustrom, J.; Antelo, M.V.; Puertas, E.B.; Rhoda, D.A.; Velandia-Gonzalez, M. (2023)

File	Contents
S1	Survey instrument (Spanish)
S2	Survey instrument (English)
S3	Percent of respondents, by demographic and COVID-19-related characteristics, region, and country of origin
